# Vertical distribution and radiological risk assessment of ^137^Cs and natural radionuclides in soil samples

**DOI:** 10.1038/s41598-019-48500-x

**Published:** 2019-08-21

**Authors:** Ayesha Y. Ahmad, Mohammad A. Al-Ghouti, Ibrahim AlSadig, Mohammed Abu-Dieyeh

**Affiliations:** 1 Department of Biological and Environmental Sciences, College of Arts and Sciences, Qatar University, State of Qatar, Doha, P.O. Box: 2713 Qatar; 2 Ministry of Municipality and Environment-Radiation and chemical protection department, P.O. Box: 36390 Doha, Qatar

**Keywords:** Environmental monitoring, Environmental impact

## Abstract

The aims of this study were to investigate the vertical distributions of natural radionuclides ^232^Th, ^226^Ra and ^40^K as well as anthropogenic radionuclide ^137^Cs in soil samples and to analyze the correlation among the radioactivity of these radionuclides and the physiochemical characteristics of soil samples namely pH, grain size, carbonate content and organic matter. Risk assessment of the radiological hazard has also been estimated. Forty-four soil samples were collected from eleven locations in Qatar at four depth levels from 0 to 16 cm. The average concentrations of ^232^Th, ^226^Ra, ^40^K and ^137^Cs in the soil depth of 16 cm were 10, 17, 201 and 4 Bq/kg, respectively, which were within the reported world mean. The external absorbed gamma dose rate, the annual effective dose, the mean radium equivalent activity, the external hazard index and the lifetime cancer risk were 22 nGy/h, 0.027 mSv/y, 47 Bq/kg, 0.125 and 0.096 × 10^−3^, respectively. These values were far below the minimum recommended international values. The level of radioactivity concentrations in the soil was affected by the physiochemical characteristics of the soil. The positive correlation with highest R^2^ value was found among the radioactivity concentrations of ^232^Th and ^40^K and the soil clay content. Total organic carbon was also positively correlated for ^226^Ra and ^137^Cs activity concentrations, whereas, carbonate content was negatively correlated with the radioactivity concentrations of ^232^Th and ^40^K. As far as soil moisture content is concerned, the positive correlation with highest R^2^ value was obtained for ^226^Ra activity concentrations.

## Introduction

Radioactivity has become an issue of major concern over the years due to its association with human health^1,2^. Natural and artificial radioactive isotopes are host in the environment. Radionuclides, with different biogeochemical processes and important movability, can influence the environment through bioaccumulation and are hazardous for the environment and human health. The radioactive isotopes in the environment cause the external radiation dose to human organisms, while the isotopes integrated by inhalation and ingestion are the origin of the internal radiation dose. Studies about dose-effect relationships of radioactive materials have helped to increase knowledge about the risks associated with radiations and have played an important role in developing radiation protection regulations.

Characteristic of soil is instantaneously concerned to the essence of life^3^. The risk from Cs-137 varies with its diffusion rates in soil. If Cs-137 migrate slowly in soil, the internal irradiation will be higher due to higher absorption by plants roots especially from the top surface of 5 cm depth. However, if Cs-137 diffuse rapidly, the external radiation will be less as in this case, the uppermost soil surface acts as a shield against radioactivity found in deeper soil layers^4^. Therefore, assessment of the diffusion of natural and artificial radionuclides in soil is important to protect human and environment^5^.

Naturally Occurring Radioactive Materials (NORM) refers to the natural origins of radiations i.e. from naturally occurring radioisotopes^6^. Natural radioactivity can result from cosmogenic radioactive isotopes which are continuously produced by the effect of cosmic radiation (e.g., ^14^C, ^3^H, ^7,10^Be, ^22^Na, ^26^Al, ^32,33^P, ^35^S, ^36^Cl, and ^39^Ar) and terrestrial radioactive isotopes^7^. Terrestrial radionuclides contain nucleogenesis or primordial natural radioactive isotopes. This can be classified into: natural radioactive decay series (^235^U, ^238^U, and ^232^Th) and the daughter nuclides with relatively long half-lives and the daughter elements of these daughter nuclides, for example, ^226^Ra, ^210^Pb, ^210^Bi, and ^210^Po. The second group is the long-life nuclei, which become stable daughter nuclides in one-step; ^40^K is the most critical radionuclide in this group. The ^40^K isotope emits gamma radiation with high energy (1.46 MeV)^1^.

Changing in the natural state of NORM causes technologically enhanced naturally occurring radioactive material (TENORM), or enhanced levels of NORM^8^. The anthropogenic activities such as coal mining, oil and gas extraction, geothermal energy production, water and wastewater treatment, application of phosphate fertilizers, uranium, thorium and copper mining can increase the level of naturally occurring radioactive particles^9^.

Artificial concentrations are the ones that results from human activities^10^. There are over 1300 artificially produced radionuclides. Due to ^137^Cs comparatively long half-life (30.17 years), considerable amounts of ^137^Cs are present in soil^11^. The main sources of Cs-137 in the environment worldwide are from nuclear atmospheric testing and regionally from the releases from the Chernobyl accident in 1986 and the Fukushima accident in 2011. The disaster in Chernobyl nuclear power station in Ukraine (on 26 of April 1986) released huge mass of radioactive materials. This accident has released up to 3.8 × 10^16^ Bq of ^137^Cs to the environment.

In Qatar, the radiological risk may arise from accidents where the sealed radiation sources are destroyed or lost and from unmanageable radioactive waste from intensive oil industry activities in oil drilling and exploitation. On the other hand, the anthropogenic radionuclide may arise from previous weapon test, nuclear accident, Gulf war and/or from progress development of nuclear industry in neighboring countries. Evaluation of radioactivity levels of several soil samples for the State of Qatar intends to aid as a reference for future radiological investigations, bioavailability assessment and divination future dose rates to save the human health and environment, emergency action management, and outline the regulatory control standards and recommendations.

The vertical distribution of ^137^Cs is tightly related to organic matter distribution. The contents of silt and clay are the second distribution factor of ^137^Cs^9^. The key factor in the fixation process is the low hydration energy of the Cs ion. The adsorption properties of organic matter and content of silt and clay minerals give the soil particles high cationic exchange capacity. ^137^Cs, which is highly reactive, will perform as a cation like K^+^, H^+^ or NH_4_^+^ as well as other competitive minerals like Na^+^, Ca^2+^ or Mg^2+ ^^12^. Thus, the vertical migration of ^137^Cs can be quicker in places with large coarse soil particles holding lower cation exchange capacity than in fine soil particles^13^. Furthermore, the soil pH also plays a major role in determining the transport of ^137^Cs within soil profile. At low pH value, more ^137^Cs is detected. Physical soil actions caused by runoff can also affect the final distribution of ^137^Cs^14,15^.

Therefore, the aims of this study were to: (i) determine the vertical distribution of ^137^Cs and natural radionuclides within a 16-cm soil depth in order to quantify spatial radioactivity variations; (ii) measure the activity concentrations of some radionuclides associates of the uranium–radium (^238^U - ^226^Ra), thorium- actinium (^232^Th - ^228^Ac) decay series, and of primordial radionuclide ^40^K as natural sources of radioactivity, and the activity concentrations of ^137^Cs as anthropogenic source of radioactivity; (iii) measure the physicochemical characteristics of the collected soils namely pH, grain size, moisture content, carbonate content and total organic carbon; (iv) assess radiological risk and concentrations; and (v) statistically analyze the correlation between physiochemical characteristic of soil namely: pH, moisture content, grain size, carbonate content and total organic carbon, with the vertical distribution of radionuclides in the soil.

## Materials and Methods

### Description of the study region

The State of Qatar has a total area of 11,437 km^2^. The geological structure of Qatar consists of a sequence of limestone, chalk, clay and gypsum^16^. Qatar topography is a rocky desert land with spread oases shaped by 850 scatter depressions. Colluvium soils, which made up of sandy clay loam, calcareous loam and sandy loam have piled up in these depressions to depths leveling from 30 to 150 cm, mainly in northern and eastern areas, covering limestone bedrock. These depression soils, that are locally named as Rauda (means garden), were preferred in the current study for the soil sampling sites due to accumulation of washout surface soil by rainfall. Rauda soil is buildup of rather bulky sediments in shallow depressions that break the topography as the result of surface water erosional from short-living channels after heavy rain^17^. The average yearly rainfall in Qatar is about 100 mm per year, mostly during autumn and winter seasons and more rainfall takes place in northern and eastern areas as compare to southern areas. Therefore, the sampling sites in current study were selected mainly from northern areas of Qatar. Summer season, which start from June to October, are described by severe heat, dryness, quite changeable humidity and unusual strong wind and sandstorms^16^.

### Sample locations

The sampling was mainly for uncultivated soils within the specified region in Qatar. The inclination of the selected regions towards the North of Qatar was due to its topography. The North of Qatar has lower altitude with scattered depressions called locally Rauda; that allows the rain to accumulate in these areas. In addition, the annual rainfall in northern of Qatar is higher than the southern, which enhanced the wet deposition in those areas. The dry deposition is also enhanced by the lower average temperature in northern areas of Qatar, whereas, in southern areas, higher temperature at the soil surface resists the dry deposition. The sampling regions were: Al-Shamal, Al-Zubara, Al-Areesh, Fwaret, Ras Lafan, Al-Guwariah, Al-Khawr, Al-Jamaliayah, Dukhan, Al-Shahaniyah and Um Al-Amad.

### Soil sample collection

Forty-four samples were collected. The samples were dug and scraped using shovel and scoop by a corer 30 cm × 30 cm. The soil was removed layer by layer at approximately depth intervals of 5 cm with a total of four different depth levels (0–1), (1–6), (6–11), (11–16) cm in each location. Accumulatively, a depth of 16 cm was used for the sampling process. As the existence of foreign materials are irrelevant for the soil samples and may give an error in the analytical results, glass pieces, twigs, stones, or leaves were removed from the soil samples. It should also be noted that the soil was undisturbed. Each layer sample was then packed into tagged polyethylene bags and sealed. The information of each sample was documented with a waterproof marker pen on each sample bag. The labels included soil information like: the region name, geographic coordinate, date, depth and ID (identified number). Three-digit serial numbers were used for numbering the collected soil samples (for example, 01.1 means (01) digit identified location Fwaret then (0.1) digit for soil of depth level 0–1 cm). The sampling tools were always cleaned during sampling to prevent soil-to-soil contamination.

The soil samples were then mixed and homogenized well and then divided into two portions for further analysis: for the radioactivity measurements with a total of 88 samples (including replication) and for the physiochemical characteristics with a total of 44 samples. The physiochemical characteristics include pH, moisture content, grain size, carbonate content and total organic carbon. Each bag was labeled with date, sample ID, location and coordinate.

### Radioactivity concentration measurements

The samples were dried in an oven at 60 °C for 24 hours in order to assure that any moisture was eliminated from the samples. To gain uniform particle sizes, a 2 mm mesh was utilized to sieve the samples. The sample was then weighed and delivered to 250 ml tagged Marinelli beakers to remove the air by tapping Marinelli beakers over a surface, then was sealed in the airtight Marinelli beakers. The samples were manually homogenized during the sieving process using the sieving pans, and then by using a stainless-steel spoon to press the soil (homogenization is blending of a soil sample to grant uniform diffusion of contaminants). Incomplete homogenization would increase the sampling error) (IAEA- 2004, 2016). Marinelli beakers were then stored and kept sealed for about one month (>7 half-lives of 222Rn) to attain radioactive secular equilibrium between 222Rn and its daughters^18^.

Radioactivity concentrations of the soil samples were measured with a high purity Germanium (HPGe) gamma-ray spectrometry system. The current system was prepared with a coaxial HPGe detector. It has a relative efficiency of 40%, an energy resolution of 1.85 keV at 1332.5 keV of ^60^Co and of 0.87 keV at 122 keV of ^57^Co, a peak-to-Compton ratio of 62:1 and operating bias voltage 4000 Vdc. After subtracting the background radiation measurement, the radionuclides were estimated at the gamma lines energies as in Table 1. ^226^Ra was determined by utilizing its progenies, ^214^Pb with energies 295.2 keV and 351.93 keV and ^214^Bi with energies 609.31 keV, 1120.29 keV, 768.36 keV 1238.11 keV and 1764.49 keV. For ^232^Th, the activity level was measured by utilizing the gamma lines 338.40 keV, 964.60 keV, 969.11 and 911.60 keV for ^228^Ac and gamma line 238.63 keV for ^212^Pb. Thus, ^226^Ra and ^232^Th activities were obtained by utilizing an average value of their progenies. In the case of ^40^K and ^137^Cs, the specific activity levels were estimated directly by their gamma lines of 1460.81 keV and 661.65 keV, respectively. The efficiency calibration aims to obtain a correlation among the absolute values of the sample activities to the detected number of counts and it related to detector type, property and geometry, as well as sample size. The properties of the detector include software which permits applying mathematical modeling for the efficiency calculation curves attributed to soil weight. Utilizing the geometry composer software, a geometry file was made for 250 mL Marinelli beaker and consequently a calibration efficiency curve was created and saved. Genie 2000 Spectroscopy Software version 3.2 allows marking the efficiency calibration using the spectrum simulation. The basis for the entire Simulator’s functionality is the “Template” file, a CAM file containing an actual spectrum. Any CAM file can be used, and the behavior of the Simulator, especially the simulated spectrum, will match that of the template file. As a result, any real spectrum can be simulated. In the end the software gives efficiency calibration values needing just to insert input data about the properties of the supposed diffusion of the radionuclides in the soil and measurements geometry.

**Table 1:** The activity concentration of radionuclides analysis from gamma ray energies of their progenies^39^.

Radionuclide	Half-life (yr.)	Gamma ray energy (keV)	Progeny radionuclide
^226^Ra	1650	295.21	^214^Pb
		351.92	^214^Pb
		609.31	^214^Bi
		1120.29	^214^Bi
		1764.49	^214^Bi
		1238.11	^214^Bi
		768.36	^214^Bi
^232^Th	1.405 × 10^10^	338.32	^228^Ac
		911.60	^228^Ac
		964.60	^228^Ac
		969.11	^228^Ac
		238.63	^212^Pb
^40^K	1.278 × 10^9^	1460.81	^40^Ar
^137^Cs	30.1	661.66	^137^Ba

The statistical uncertainty contributed with the total counts in the peaks, whereas the systematic uncertainty is contributed to the combined uncertainty of the efficiency calibration^12^. The uncertainties were estimated utilizing error propagation, accounting for relative standard uncertainties of the sample weight, net peak area, full energy peak efficiency, half-life of the radionuclide and emission probability^19^.

The software used for analysis and reduction of the gamma-ray spectra was Genie 2000 Spectroscopy Software. The radioactivity concentration of the radionuclide established in the soil samples was measured by utilizing Eq. (1) and represented in unit of Bq/kg:

1 $${\rm{Radioactivity}}={{\rm{C}}}_{{\rm{net}}}/({\rm{\gamma }}\times \varepsilon \,({\rm{E}}\,{\rm{\gamma }})\times {\rm{m}}$$

where C_net_ is the net peak count rate, γ is the absolute gamma decay intensity for the specific energy photopeak, ε(Eγ) the absolute photopeak efficiency of the germanium detector at this energy and m is the mass of the sample in kg.

### Physiochemical characterization measurements

Various physiochemical characterization for the soils were measured; namely pH, grain size measurement by laser diffraction, moisture content measurement, carbonate measurement, and total organic carbon measurement.

## Results and Discussion

### Physiochemical characterizations results

**Figure 1:**
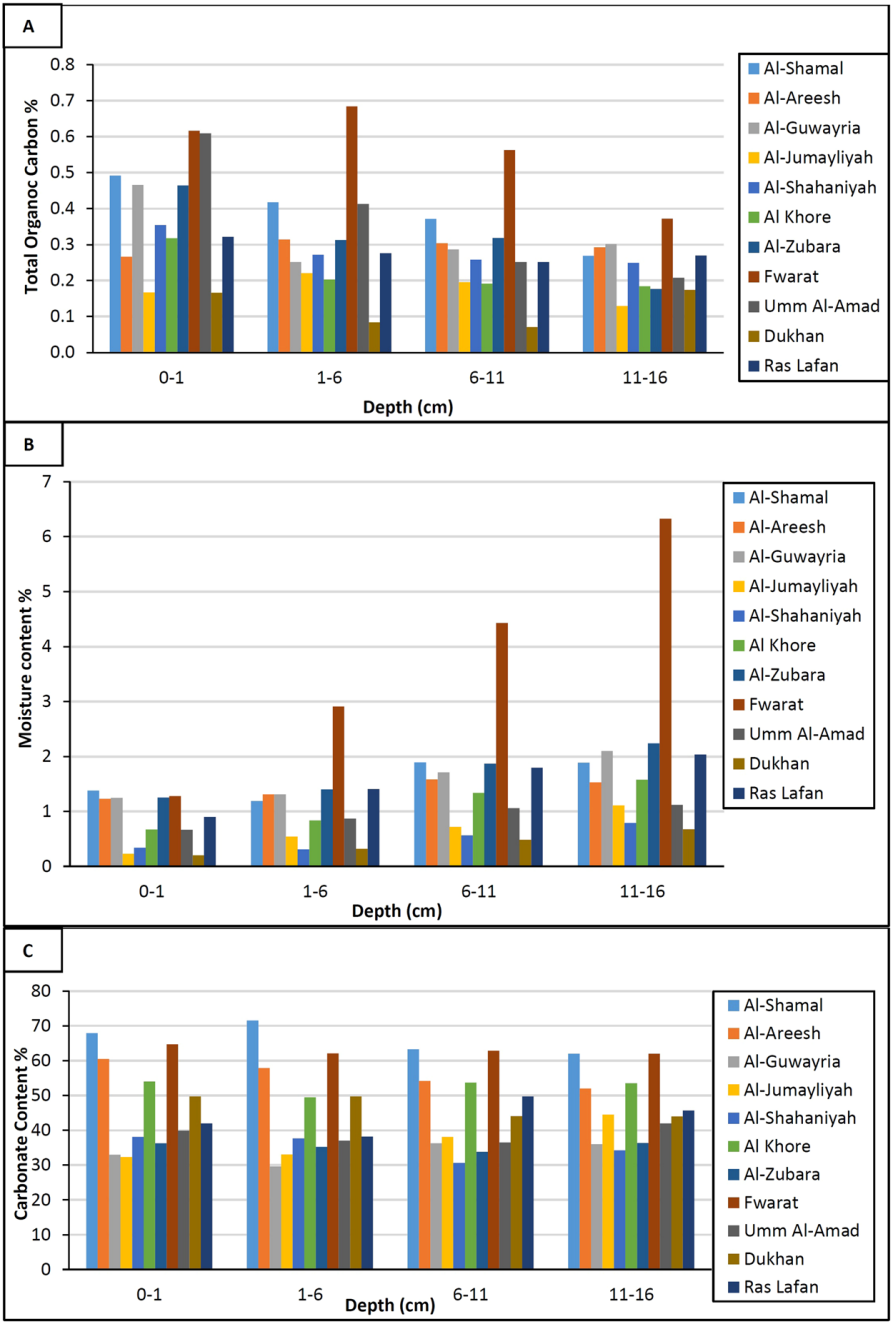
(**A**) % TOC content, (**B**) % Moisture content, (**C**) % Carbonate content – collected from different soil samples in Qatar.

The vertical distribution of radionuclides depends greatly on the soil characteristics^20^. Radioactivity in soil declines by leaching of water, attenuation by enhanced porosity and by supplementary water and organic matter and rises by sorption and precipitation of radionuclides from incoming water^5,21^. Figure 1 illustrates the vertical distribution of total organic carbon (TOC%) for soil samples in each depth level for 11 locations in Qatar. TOC gives an indicator about the organic matters in soil samples. Figure 1 shows that the organic matter is very low for all soil samples mainly due to low vegetation cover; it varies from 0.08% to 0.68%. Because of the high cationic exchange capacity of the organic matter, the results of low organic matter may lead to assumption that adsorption in soil samples may be controlled mainly by an ion exchange mechanism with the clay minerals^15^. Organic matter is of great importance because it tends to form soluble or insoluble complexes with radionuclides, which may then transport throughout the profile or retain within the soil^22^. Generally, the top soil samples contain higher organic matter content than the deeper soil samples due to the dominance of pedogenetic processes in upper soil layer. The highest organic matter was in Fwaret soil samples in the top layer from 0–1 cm and 1–6 cm. This was due to geological structure of soil in Fwaret called Sabkha with deposits of saline and gypsiferous sand and silt flat with halophytes plants. Sabkha contained elevated radioactivity concertation of ^226^Ra^23^.

The moisture content of the soil samples in four depth layers is also presented in Fig. 1. It shows that the moisture content of all soil samples was very low which could be due to the season in which sampling was done. It was conducted during summer season where the temperature was from 44–47 °C; the moisture content percentages varied from 0.228% to 6.32%. Generally, the topsoil receives direct sunshine, and therefore the topsoil samples contained lower moisture content than the deeper soil samples. The highest moisture content was in the Fwaret soil samples in the deepest layer from 11–16 cm. The lowest moisture content was in Dukhan soil samples collected from deeper depth i.e. 6–11 cm. According to literature survey, soil moisture content generally has a direct impact on redox potential and radionuclide speciation^22^. Low moisture content would lead to less hydrophilic radionuclide due to decrease in solubility. In addition, radioactive gases which are not fully trapped in soil can also easily be dispersed into the atmosphere; such as: ^226^Ra and ^224^Ra that decay to ^222^Rn and ^220^Rn gases, respectively^3^.

The pH values of the soil samples were in the range from 7.2 to 8.6, which means that the soil was mostly alkaline. According to literature, pH has a strong influence on the mobility of radionuclides that may precipitate some soil components such as carbonates^18^. At high pH, various precipitates would be formed such as carbonate and hydroxyl, phosphate or sulfide ions complexes. These insoluble precipitates reduce the availability of radionuclides in upper soil surface. While at low pH, radionuclide cations may have displaced by H^+ ^^24^. Actinides and other redox-sensitive elements are mobile in oxidizing conditions and more strongly immobilized in reducing environments. So, any reducing environment in each site can play as an efficient radionuclide^25^. In summary, Fe is precipitated as oxyhydroxide under alkaline pH, which has the high affinity to scavenge other metals^26^. Cationic complexes are also abundant in slightly alkaline pH if the soil is rich in organic matter due to the high cationic exchange capacity of the organic matter. However, organic matter is not present in high quantity in Qatar’s soil^17^. At high pH, the negative charge dominated the surface of the functional group of humic matter^24^.

Concerning the particle size of soil, all samples were mainly comprised of sand except for Al-Guwayria soil sample, which contained silt. According to the literature, the concentration of clay minerals can affect the distribution of radionuclide bearing primary minerals and consequent radionuclide activity. Soil with sand particles will lead to lower adsorption of radionuclide to soil particles than soil with high clay and silt content^5^. Sand particles have lower adsorption due to lower surface area and ion exchange capacity (between aluminum silicate (anions) that readily adsorb radionuclide cations on their surface) than clay and silt^7^.

Carbonate content percentage ranged from 29.59% to 71.52% (Fig. 1). According to the literature, high carbonate content may influence processes such as interaction with C-bearing minerals and adsorption of radionuclide to soil particles and consequently concentration of radionuclide due to the competition for ion exchange of radionuclide ions with Ca^2+^ on soil particles surface^3^. At alkaline environment, carbonate ligands become the major complexing species in solution^24^.

### Radioactivity concentration results

**Figure 2:**
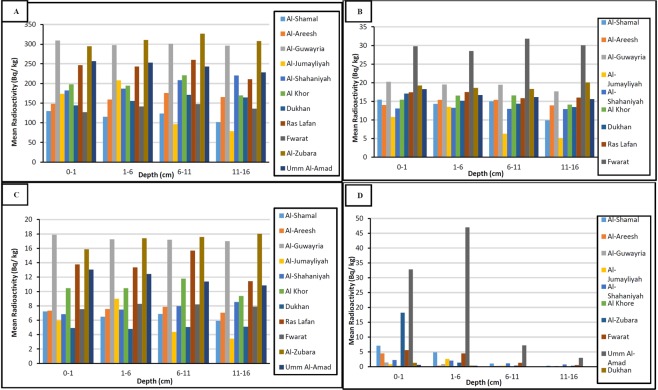
(**A**) ^40^K Radioactivity concentration (Bq/kg), (**B**) ^226^Ra Radioactivity concentration (Bq/kg), (**C**) ^232^Th Radioactivity concentration (Bq/kg), and (**D**) ^137^Cs Radioactivity concentration (Bq/kg) in the soil samples.

The radioactivity levels of the radionuclide in the soil samples were determined by Canberra Genie 2000 spectroscopy software. The weighted mean radioactivity level of ^226^Ra, ^232^Th, ^40^K and ^137^Cs in the four soil depth levels (0–1 cm), (1–6 cm), (6–11 cm), and (11–16 cm) for each location site are shown in Fig. 2. The Figure illustrates that the radioactivity concentration levels of the natural radionuclides ^226^Ra, ^232^Th and ^40^K vary substantially in each location site depending primarily on the concentration of radionuclides in bedrocks from which the soil originates. Most limestones have relatively little radium concentration^27^. Besides, the levels of ^226^Ra, ^232^Th and ^40^K in the soil might be affected by the physiochemical characteristic of the soil. Whereas, the activity concentration of the anthropogenic radionuclide ^137^Cs varied in each location site due to the variation in the metrological factors at the time of deposition, as well as to the physiochemical characteristics of the soil^28,29^.

In general, it can be noticed that the radioactivity concentration levels were very low. The IAEA -suggests that it is not required to assign regulatory control for activity levels lower than 1 Bq/g. Though the activity levels at one or above can be exempt from regulatory controls if occupational exposure is established to be less than 1 mSv/y^30^.

Figure 2 illustrates the vertical distribution of ^40^K radioactivity concentration of four depth levels in different sample location sites. The highest ^40^K radioactivity concentration was in Al-Zubara (327 ± 14 Bq/kg) in depth soil of (6–11 cm), whereas, the minimum ^40^K radioactivity concentration was in Al-Jumayliah (79 ± 3 Bq/kg) in depth soil of (11–16 cm). The high concentration of ^40^K could be attributed to high clay content; consequently, low radioactivity concentration could be correlated to high sand content. ^40^K is part of a clay minerals component rather than organic matter, and its mobility is controlled by the solubility in the soil^28^.

Figure 2 shows the vertical distribution of ^226^Ra radioactivity concentration of four depth levels in different sample location sites. Commonly, limestones have relatively low radium contents^31^. The highest ^226^Ra radioactivity concentration was in the Fwaret soil sample (32 ± 1 Bq/kg) in depth soil of (6–11 cm). The minimum ^226^Ra radioactivity concentration was in Al-Jumayliah (5 ± 2 Bq/kg) in depth soil of (11–16 cm). The result of high ^226^Ra radioactivity concentration in Sabkha soil from Fwarat in the current study is in line with reported elevated radioactivity concentration of ^226^Ra in Dukhan Sabkha in previous research^23^. In this study, the elevated ^226^Ra radioactivity concentration can be attributed to radium’s co-precipitation with strontium, barium or calcium in the celestite crystal of the Sabkha. In addition, it may also be correlated to high moisture content in Sabkha soil as ^226^Ra is highly soluble^32^.

Figure 2 illustrates the vertical distribution of ^232^Th radioactivity concentration of four depth levels in different sample location sites. The highest ^232^Th radioactivity concentration was in Al-Zubara soil samples (18 ± 1 Bq/kg) in depth soil of (11–16 cm), whereas, the minimum ^232^Th radioactivity concentration was in Al-Jumayliah (3 ± 0.1 Bq/kg) in depth soil of (11–16 cm). Al-Zubara soil samples are clayey silty sand and have large carbonate content, which could be correlated to high ^232^Th concentration. It was observed from Fig. 2 that the radioactivity concentration levels of ^226^Ra and ^232^Th decay products as well as ^40^K in the surface and deep soil samples were similarly distributed, which is consistent with results of other authors in Qatar^23^ and in the worldwide^29^.

The minimum detectable activity (MDA) for the counting time of 20,000 s was 0.03 Bq/kg for 137Cs. 137Cs concentrations per unit mass in Bq/kg dry weight. The average value of the MDA for 226Ra, 232Th, 40 K and 137Cs was established as 0.05, 0.03, 0.16 and 0.01 Bq/kg, respectively.

^137^Cs is detected in trace amounts. Mainly any detected values higher than MDA in this study may result from the fallout of ^137^Cs from severe nuclear reactor accidents and atmospheric nuclear weapons tests. Figure 2 illustrates the vertical distribution of ^137^Cs radioactivity concentration of four depth levels in different sample location sites. It was clear that ^137^Cs radioactivity concentration was high in the topsoil layers and less in the deeper layers. ^137^Cs radioactivity concentration in surface soil depth at (0–1 cm) and (1–6 cm) in all soil samples were found to be higher than deeper depth at (6–11 cm) and (11–16 cm). Thus, it is present as a permanent source of external gamma dose for several years until it totally decays, however, contamination of the food chain and raise of the internal dose from the human ingestion is very unlikely. The highest ^137^Cs radioactivity concentration was in Umm Al-Alamaad (47 ± 2 Bq/kg), while the lowest ^137^Cs radioactivity concentration were in AL-Khor, Ras Lafan, Dukhan and Al-Zubara which were in minimum detectable activity range. Some of the relatively high concentrations of ^137^Cs can be attributed to rain washouts in these depression sample locations that have lower height relative to the surroundings. The high ^137^Cs radioactivity concentration can also be correlated to high total organic carbon in these soil samples.

**Figure 3:**
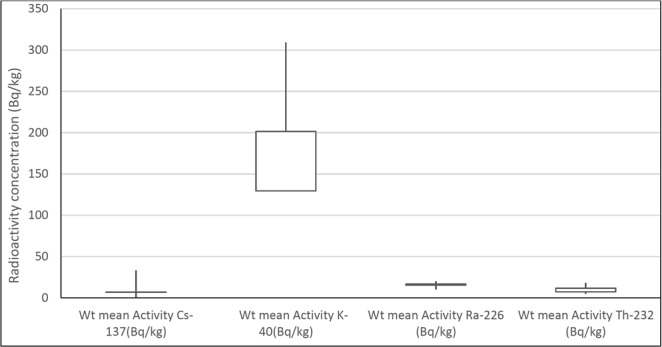
Comparison of the mean radioactivity concentration values of ^226^Ra, ^232^Th, ^40^K, and ^137^Cs (Bq/kg) of soil samples in Qatar.

The average concentrations of ^232^Th, ^226^Ra, ^40^K and ^137^Cs in the 16-cm depth soil were 10, 17, 201 and 4 Bq/kg, respectively. It was noticed from Fig. 3 that in all sampling sites, the average radioactivity level was of the order ^40^K > ^226^Ra > ^232^Th > ^137^Cs. The radioactivity concentration levels of ^40^K were the dominant gamma radioactivity source in the soil. It is well known that ^40^K in the earth’s crust is of the order of percentage while ^226^Ra and ^232^Th are in ppm level. ^40^K is a common primary weathering product^5^.

### Comparison of activity concentrations with other studies

Table 2 represents a comparison of weighted mean radioactivity concentration of ^226^Ra ^232^Th, ^40^K, and ^137^Cs with data of -previous studies in different countries. The average radioactivity concentrations for the for the 16 cm depth in Qatar soil samples are 17, 10, 201 and 4 Bq/kg for ^226^Ra, ^232^Th, ^40^K and ^137^Cs, respectively, which were below the world averages concentration. It was clear that the radioactivity concentrations were consistent with the previous reported studies in Qatar. The average concentrations of ^232^Th, ^226^Ra, ^40^K and ^137^Cs for the 16 cm depth in Qatar soil were 9, 17, 204 and 6 Bq/kg, respectively^23^. The obtained results were generally comparable to the literature data acquired from other countries, and all are within the worldwide average concentration except in Nigeria, India and Yemen, which are higher than the worldwide mean concentration due to geology and bedrocks of these two countries. The mean radioactivity concentration was found to be lower than the obtained values of those reported in the USA, Turkey, Jordan and Berlin. It also was comparable to the mean concentration published in neighboring countries in Iraq, Kuwait and Saudi Arabia. The variation could mainly be due to the different geology as well as the difference in sampling depth, physiochemical soil characteristic and metrological factors at time of the deposition.

**Table 2:** Comparison of the worldwide weighted mean radioactivity concentration of ^238^U, ^226^Ra, ^232^Th, ^40^K, and ^137^Cs (Bq/kg) with the current study.

Country	Depth (cm)	Weighted	Mean	Radioactivity	Concentration (Bq/kg)
		^226^Ra	^232^Th	^40^K	^137^Cs
Worldwide value^27^		32	45	412	51
Current study	**0–16**	**17**	**10**	**201**	**4**
Qatar^23^	5–15	17.22	9.3	204	5.8
Saudi Arabia^39^	10	22.7	14.8	392	<MDA
Kuwait^28^	5–25	23.9	14.1	368	<MDA
Iraq^43^	0–15	—	6.2	293	—
Turkey^46^	15–20	38.1	39.3	375.3	12.1
Jordan^42^	1	—	26.7	291.1	—
India^26^	5–15	42.90	72.96	572.40	3.10
Nigeria^47^	0–40	75.25	56.60	811.85	0.59
Yemen^48^	2	44.4	58.2	822.7	4.8
Berlin^31^	0–5	27.1	34.3	370.5	18.6
USA (Texas)^11^	0–10	33.7	—	299.7	3.7

MDA: minimum detectable activity.

### Statistical analysis and correlation of physiochemical characteristic of soil with radioactivity concentration

**Figure 4:**
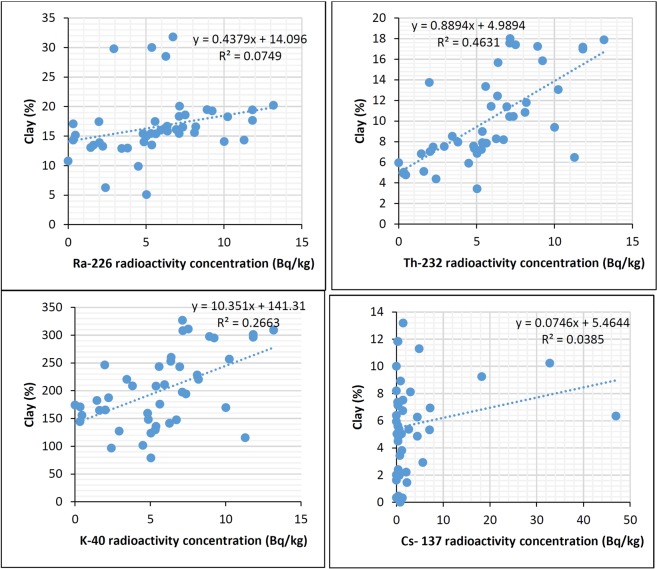
Correlation between clay content and radioactivity concentration of ^226^Ra, ^232^Th, ^40^K, and ^137^Cs (Bq/kg) in soil samples.

**Figure 5:**
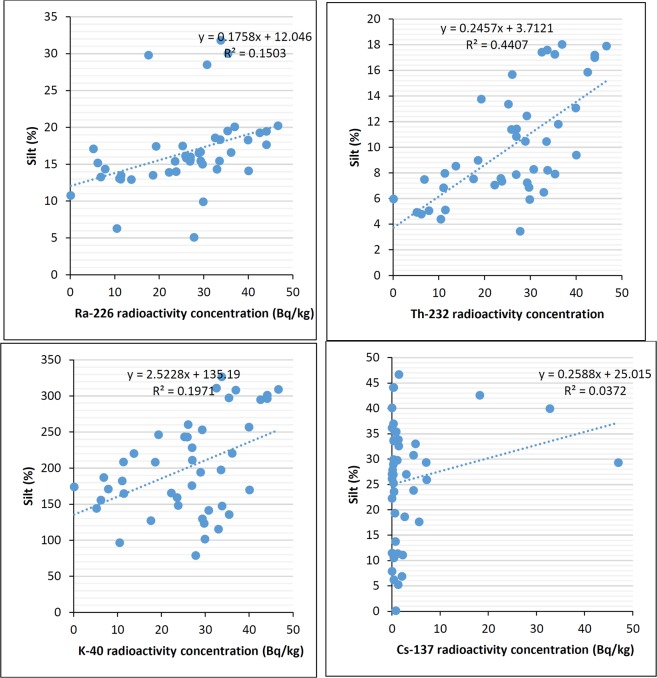
Correlation between silt content and radioactivity concentration of ^226^Ra, ^232^Th, ^40^K, and ^137^Cs (Bq/kg) in soil samples.

Statistical analysis was conducted using the analysis of variance (ANOVA) to ascertain whether there are significant differences in the variables analyzed. According to ANOVA analysis, there was no significant difference between ^226^Ra ^232^Th, ^40^K and ^137^Cs radioactivity concentration in the soil samples, which is consistent with the findings of previous studies^4,30^. The examination of the relationships among radionuclides and the physio-chemical characteristics of the soil was performed through principle component analysis (PCA) and Pearson correlations. The Pearson correlation coefficient was used to measure the strength of the linear relationships between physiochemical properties; namely clay content, silt content, sand content, carbonate content, total organic carbon, and moisture content with radioactivity concentrations. The obtained results are presented in Figs 4 and 5.

Comparison of ^226^Ra ^232^Th, ^40^K and ^137^Cs radioactivity concentrations between different particle sizes of soil confirmed that the presence of clay positively correlates to radionuclides content in the soil, which is in line with the findings of^33^. The best positive correlation was obtained between clay content, and ^232^Th and ^40^K activity concentrations with R^2^ = 0.46 and 0.26, respectively (see Fig. 4). This was expected as natural radionuclides are adsorbed to clay surface due to larger adsorbed surface area and lattice defects, as well as the associated clay minerals, which increases the ion exchange capacity with the radionuclides^18^. Furthermore, most ^40^K is adsorbed on the cation exchange sites of clay minerals. The positive correlation was also obtained between silt content, and ^232^Th and ^40^K activity concentrations with R^2^ = 0.44 and 0.19, respectively (see Fig. 5). However, there was a negative correlation found between the radioactivity concentration and the sand content. The negative correlations with highest R^2^ values were obtained between sand content and ^232^Th and ^40^K activity concentrations with R^2^ = 0.45 and 0.21, respectively (see Supplementary Materials). Carbonate content was negatively correlated with ^232^Th, ^40^K and ^137^Cs, which is similar to the result obtained by^18^. The anions carbonate and bicarbonate can react with the radionuclides forming complexes which are either not adsorbed at all or only slightly adsorbed onto clays. The carbonate bearing cations, on the other hand, compete with the radionuclides for available adsorption sites on the sorbing mineral surfaces, and may thereby enhance nuclide migration if present in large concentrations^34^. The negative correlation was also obtained between carbonate content with ^40^K and ^232^Th activity concentrations with R^2^ = 0.47 and 0.22, respectively (see Supplementary Materials). Total organic carbon as an indicator for organic matter was found to be positively correlated with ^226^Ra and ^137^Cs activity concentrations with R^2^ = 0.41 and 0.187, respectively (see Supplementary Materials) which is similar to conclusions of^29^. According to the literature, the soil organic matter contains chains of carbon atoms, containing polar and/or ionized surface functional groups, like OH^–^ and COOH^–^, and generally, radionuclides strongly adsorbed to these functional groups and form stable complexes^19^. The strong correlation was not found between particle size and carbonate content of soil with ^137^Cs radioactivity, which is consistent with the previous studies^4,15,35,36^. Concerning the moisture content, the positive correlation was obtained with ^226^Ra activity concentrations with R^2^ = 0.44 (see Supplementary Materials), which agreed with the study of^32^. The high radioactivity of ^226^Ra could be due to its high solubility.

**Figure 6:**
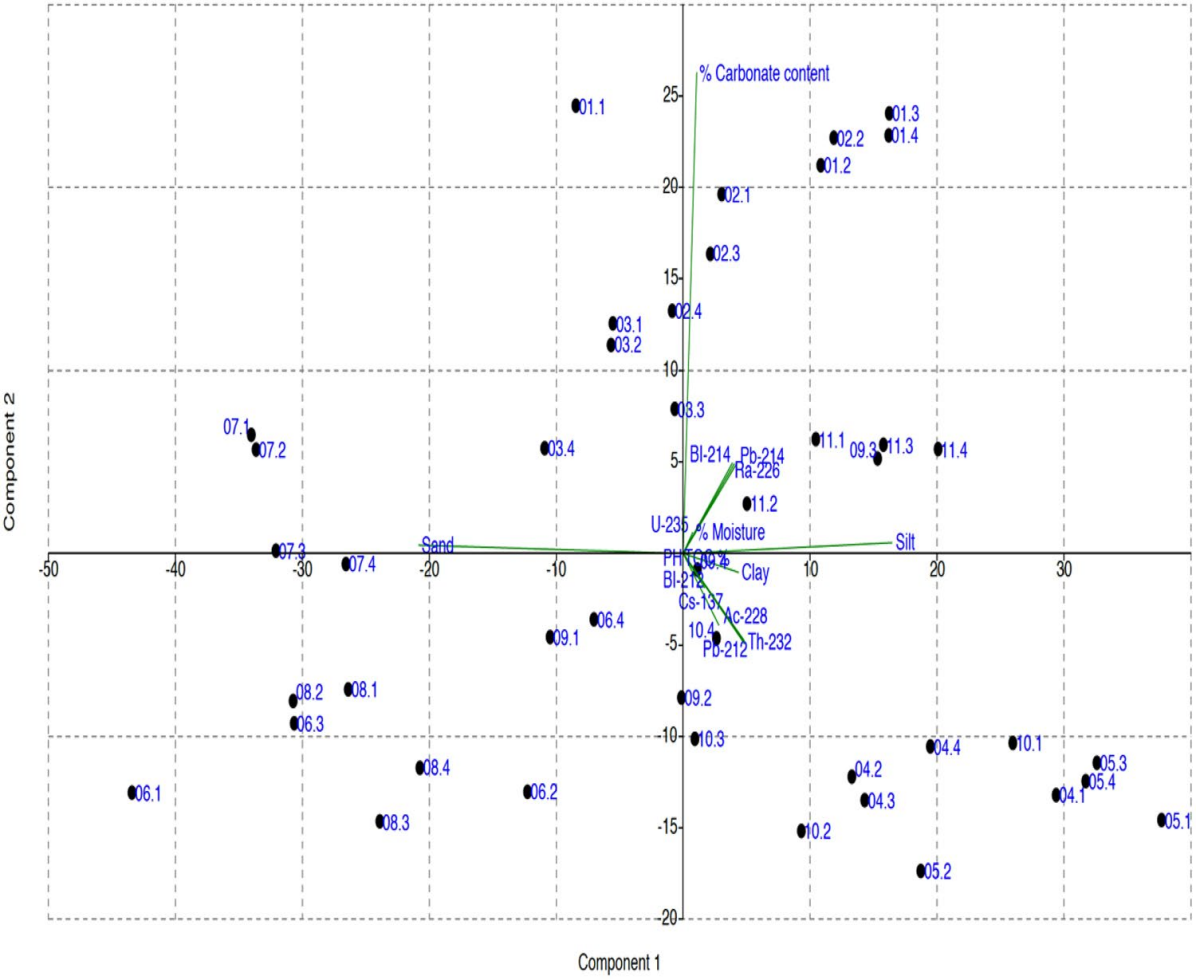
Principle component analysis for the correlation between physiochemical characteristics and radioactivity concentration of ^226^Ra, ^232^Th, ^40^K, and ^137^Cs (Bq/kg) of soil samples.

Figure 6 shows the principle component analysis (PCA) for the correlation between physiochemical characteristics and radioactivity concentration of ^226^Ra, ^232^Th, ^40^K, and ^137^Cs (Bq/kg) of the soil samples. PCA explained a linear combination of the original variables. Figure 6 shows a well correlation between silt and moisture content with the radioactivity concentration of ^226^Ra in the first component. The second component was well correlated ^232^Th and ^137^Cs concentration to the total organic carbon, pH and clay content in soil.

### Radiological risk assessment

By applying these factors, the total absorbed gamma dose rate (D) in air at 1 m above the ground level was determined as given in the Eq. (2) below provided by the UNSCEAR report^6^.

2 $${\rm{D}}\,({\rm{nGy}}/{\rm{h}})=0.462\,{{\rm{A}}}_{{\rm{Ra}}}+0.604\,{{\rm{A}}}_{{\rm{Th}}}+0.0417\,{{\rm{A}}}_{{\rm{K}}}$$

where A_Ra_, A_Th_ and A_K_ are the activity concentrations of ^226^Ra, ^232^Th, and ^40^K in Bq/kg, respectively. the coefficient 0.462 is given for the complete 238U-series (thereby including gamma radiation from the 238U-daughters 234-Th and 234 m-Pa, although the contribution to the dose from these daughters is relatively low).

Accordingly, the calculated D values from Eq. (2) are provided in Table 3. The estimated mean absorbed doses in outdoor were appeared to be in the range 15.13–32.31 nGy/h. Accordance to UNSCEAR, the average D value in the world is 51 nGy/h^37^. The result obtained by this study of average absorbed dose for the soil samples in outdoor was 22 nGy/h, which is far below the average dose value in the world.

**Table 3:** Radium equivalent activity (Bq/kg), absorbed gamma radiation dose rate in air (nGy/h), annual effective dose (mSv/y), external radiation hazard index (H_ex_) and lifetime cancer risk (LTCR) with the current soil samples in Qatar.

Soil Sample Location	Dose rate (D) (nGy/h)	Radium equivalent activity (Ra_eq_) (Bq/kg)	_External hazard index_ (H_ex)_	Annual effective dose equivalent (AEDE) (mSv/y)	Lifetime Cancer Risk (LTCR) × 10^−3^
Fwaret	25	52	0.14	0.03	0.105
Al-Shamal	15	32	0.086	0.018	0.065
Al-Areesh	18	38	0.102	0.022	0.078
Al-Zubara	32	68	0.182	0.039	0.139
Al-Guwayria	32	67	0.181	0.039	0.138
Al-Jumayliyah	13	28	0.075	0.016	0.057
Dukhan	17	34	0.092	0.020	0.071
Al-Shahaniyah	19	39	0.106	0.023	0.082
Ras Lafan	30	64	0.171	0.037	0.137
Umm Al-Amad	25	53	0.142	0.031	0.108
Al-Khor	22	46	0.123	0.026	0.941

Radium equivalent activity (Ra_eq_) is the widely used parameter to assess the gamma ray hazards^38^, which is estimated on the assumption that 370 Bq/kg ^226^Ra or 260 Bq/kg ^232^Th or 4810 Bq/kg ^40^K yield the same gamma dose rate. The Ra_eq_ of the sample in (Bq/kg) can be achieved utilizing the Eq. (3)^39^:

3 $${{\rm{Ra}}}_{{\rm{eq}}}={{\rm{A}}}_{{\rm{Ra}}}+1.43\,{{\rm{A}}}_{{\rm{Th}}}+0.077\,{{\rm{A}}}_{{\rm{K}}}$$

where A_Ra_, A_Th_, and A_K_ are the activity concentrations of ^226^Ra, ^232^Th, and ^40^K in Bq/kg of the soil sample, respectively.

Ra_eq_ values estimated from Eq. (3) are given in Table 3. The calculated range of radium equivalent activity was established to be 31–67 Bq/kg. Accordingly, the calculated mean radium equivalent activity was established to be 47 Bq/kg. The recommended maximum value of Ra_eq_ is 370 Bq/kg^40^. All estimated values of Ra_eq_ in this study are much lower than the recommended value.

The annual external effective dose rate (AEDE) was estimated from Eq. (4)^41^

4 $${\rm{AEDE}}\,(\mu \mathrm{Sv}/{\rm{y}})={\rm{D}}\,({\rm{nGy}}/{\rm{h}})\times 8760\,({\rm{h}}/{\rm{y}})\times 0.2\times 0.7\,({\rm{Sv}}/{\rm{Gy}})\times {10}^{-3}$$

where 0.2 is the outdoor occupancy factor used for the fraction of the time spent by a person, implying that 20% of time is spent out- doors^27^; However without including spent indoors (with radiation through the building walls and radiation from the building material itself) a possible underestimation of the total external dose and the associated risk. 8760 h is the time for one year and 0.7 Sv/Gy is the conversion coefficient factor from gamma absorbed dose rate in air outdoors D, which converts the absorbed dose rate in air to human effective dose to effective dose received by adults.

The average global effective dose rate to members of public from soil with weighted mean activity concentrations of 33 Bq/kg, 32 Bq/kg, 45 Bq/kg, and 420 Bq/kg for ^238^U, ^226^Ra, ^232^Th, ^40^K, respectively is 0.460 mSv/y^27^. AEDE values estimated from Eq. (4) are shown in Table 3. The estimated range of annual mean effective dose equivalent was established to be 0.039–0.018 mSv/y. The calculated average annual mean effective dose equivalent was found to be from 0.027 mSv/y. This value is also far below the world average value of 0.46 mSv/yr^42^.

H_ex_ and H_in_ are the radiation hazards indices and are defined as the external hazard indices. These show the external exposure to gamma radiation from the studied sand and sediment samples, and internal hazard index due to internal exposure to gamma radiation from ingestion food or inhalation. Both must be less than unity and are given by Eqs (5 and 6)^38^:

5 $${{\rm{H}}}_{{\rm{ex}}}={{\rm{A}}}_{{\rm{Ra}}}/370+{{\rm{A}}}_{{\rm{Th}}}/259+{{\rm{A}}}_{{\rm{K}}}/4810$$

6 $${{\rm{H}}}_{{\rm{in}}}={{\rm{A}}}_{{\rm{Ra}}}/185+{{\rm{A}}}_{{\rm{Th}}}/259+{{\rm{A}}}_{{\rm{K}}}/4810$$

where A_Ra_, A_Th_, and A_K_ are the activity concentrations of ^226^Ra, ^232^Th, and ^40^K in Bq/kg of the soil sample, respectively.

In this study, H_ex_ was estimated only taking into consideration that the external hazard, which is caused by gamma rays, corresponds to a maximum equivalent activity of 370 Bq/kg, 259 Bq/kg and 4810 Bq/kg for ^226^Ra, ^232^Th, and ^40^K respectively. H_ex_ values estimated from Eq. (5) are shown in Table 3. The calculated range of H_ex_ was found to be 0.083–0.181. The maximum external hazard index should be less than unity^43^. From Table 3, it is concluded that all estimated values are lower than one.

Lifetime cancer risk (LTCR) caused by the annual effective dose rate was calculated utilizing Eq. (7)^44^:

7 $${\rm{LTCR}}={\rm{AEDE}}\,(\mu {\rm{Sv}}/{\rm{y}})\times {\rm{AL}}\times {\rm{RF}}$$

where external exposure (AEDE) is the annual effective dose rate, AL is the average life time (70 years) and RF is the risk factor (0.05).

The calculated values of LTCR are presented in Table 3. The values of LTCR varied from 0.064 × 10^−3^ to 0.139 × 10^−3^ with an average of 0.096 × 10^−3^ which is much less than the world average of 0.299 × 10^−3^ ^39^. From the results of radiation hazard indices in all soil samples in Qatar, we can conclude that the radiological risk for soil samples in the current study was insignificant due to the low values of calculated D, AEDE, Ra_eq_, H_ex_ and LTCR in all samples under investigation.

**Figure 7:**
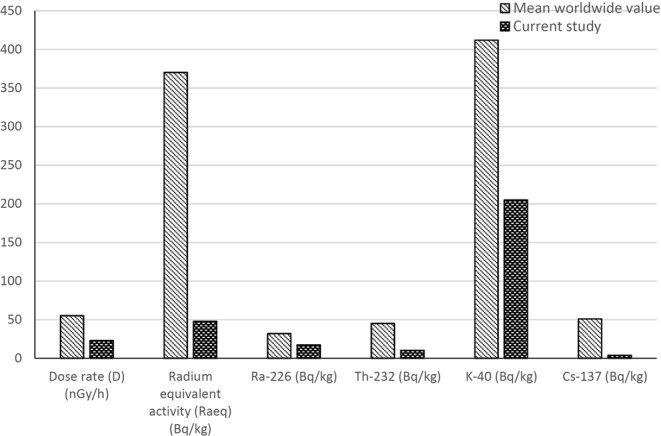
Comparison of the mean radioactivity concentration values of ^226^Ra, ^232^Th, ^40^K, and ^137^Cs (Bq/kg), the gamma dose rate (D) (nG/h), the radium equivalent (Ra_eq_) (Bq/kg) of soil samples in Qatar with worldwide permissible values.

The mean values of radium equivalent activity (Ra_eq_), the total absorbed dose rate in air 1 m above the ground (D), the annual effective dose (AEDE) and the external hazard index (H_ex)_ in this study were compared with the other global measurements for different countries of the world as given in Table 4. From Table 4, it can be noticed that the calculated D, AEDE, Ra_eq_, H_ex_ values were found to be far below the allowed limits, and all other countries indices are within the limits of^6^. The obtained radiological indices results were strongly agreed with previous results in Qatar^37^. In addition, it is shown that the radiological indices in the current study were established to be lower than the calculated values of those reported in other countries. Figure 7 shows the comparison of the mean radioactivity concentration values of ^226^Ra, ^232^Th, and ^40^K and ^137^Cs (Bq/kg), the gamma dose rate (D) (nG/h), the radium equivalent (Raeq) (Bq/kg) of soil samples in Qatar with worldwide permissible values. It is clearly indicated that results obtained from the current study were well below worldwide average permissible values.

**Table 4:** Comparison of the worldwide mean values of radium equivalent activity (Bq/kg), absorbed gamma radiation dose rate in air (nGy/h), annual effective dose (mSv/y), and external radiation hazard index (H_ex_) with the current study.

Country	Dose rate (D) (nGy/h)	Radium equivalent activity (Ra_eq_) (Bq/kg)	_External hazard index_ (H_ex)_	Annual effective dose equivalent (AEDE) (mSv/y)
worldwide average values^6^	55	370	≤1	1
Current study	**22**	**47**	**0.125**	**0.027**
Qatar^37^	24.2	50.4	0.1	0.029
Saudi Arabia^39^	37.2	74.1	0.02	0.04
Iraq^43^	23.27	46.82	0.116	0.132
Kuwait^40^	24.65	50.72	0.14	0.03
Jordan^42^	51.5	103.1	0.28	—
Turkey^36^	45	96	0.26	0.056
Nigeria^47^	86	86.32	—	0.147
Yemen^48^	89.45	191	0.52	—
Berlin^31^	51	62	—	—
USA (Texas)^11,49–53^	48.4	102.4	0.3	0.059

## Conclusion

In this current study, the activity concentrations of ^226^Ra, ^232^Th, ^40^K and ^137^Cs of the 44 soil samples collected from Qatar (Al-Shamal, Al-Zubara, Al-Areesh, Fwaret, Ras Lafan, Al-Guwariah, Al-Khawr, Al-Jamaliayah, Dukhan, Al-Shahaniyah, Um Al-Amad) were measured utilizing high purity germanium (HPGe) gamma ray spectrometry. The average activity concentrations of ^226^Ra, ^232^Th, ^40^K and ^137^Cs were lower than the average values of the earth’s crust. The average concentrations of ^232^Th, ^226^Ra, ^40^K and ^137^Cs in the 16-cm depth soil were 10.08, 16.6, 200.63 and 3.57 Bq/kg, respectively. The external absorbed gamma dose rate (D), the annual effective dose, the mean radium equivalent activity (Ra_eq_), the external hazard index (H_ex_) and in and Lifetime cancer risk (LTCR) were evaluated as 22.37 nGy/h, 0.027 mSv/y, 46.58 Bq/kg, 0.12 and 0.096 × 10^−3^, respectively. The results of the radioactivity concentration indices indicated that all soil samples are complying with the exemption annual dose criterion of 1 mSv. Calculated values for the absorbed dose rate and annual effective dose from ^232^Th, ^226^Ra, ^40^K and ^137^Cs were established to be well below the global average values, consequently pointing towards low radiation exposure to the citizenry. The mean radium equivalent activity for these radionuclides in soil samples was found to be far below the permitted value and the external radiation hazard index was also below unity. In view of the current study, the radiological impacts of radionuclides are negligible compared to the world average values^6,45^.

Uniform vertical distributions of natural radionuclides ^226^Ra, ^232^Th, and ^40^K were found in the uppermost 0–16 cm of soil. Whereas, ^137^Cs radioactivity concentration was higher at 0–6 cm than deeper depth 6–16 cm, this shows that ^137^Cs migrates very slowly in undisturbed soil.

Based on analytical results of Pearson correlation coefficient, the radioactivity concentrations were affected by the physiochemical characteristics of soil. The best positive correlation was observed between clay content, and ^232^Th and ^40^K activity concentrations and carbonate content was negatively correlated with ^232^Th, ^40^K and ^137^Cs. Total organic carbon was positively correlated with ^226^Ra and ^137^Cs activity concentrations. Concerning the moisture content, the best positive correlation was obtained with ^226^Ra activity concentrations.

## References

[1] Kónya, J. & Nagy, N. M. *Nuclear and radiochemistry* (London: Elsevier, 2012).

[2] L’Annunziata, M. F. Raddioactivity (Second Edition). *Introduction and History, from the Quantum to Quarks*. *B*.*V*. ISBN: 978-0-444-63489-4 (Elsevier, 2016).

[3] Baskaran, M. *Handbook of environmental isotope geochemistry* (Heidelberg: Springer, 2011).

[4] Belivermis M (2012). Vertical distributions of ^137^Cs, ^40^K, ^232^Th and ^226^Ra in soil samples from Istanbul and its environs, Turkey. Radiation Protection Dosimetry..

[5] Guagliardi I (2016). Effects of source rocks, soil features and climate on natural gamma radioactivity in the Crati valley (Calabria, Southern Italy). Chemosphere..

[6] UNSCEAR (2008). United Nations Scientific Committee on the Effects of Atomic Radiation. Report to the General Assembly United Nations, New York. Annex B..

[7] Bryan, J. C. Introduction to nuclear science. *Boca Raton* (CRC Press, 2009).

[8] Michalik B, Brown J, Krajewski P (2013). The fate and behaviour of enhanced natural radioactivity with respect to environmental protection. Enviro. Impact Assess. Rev..

[9] Walther C., Gupta D. *Radionuclides in the Environment* 61–80 (Springer, Cham, 2015).

[10] Velzen, L. V. *Environmental Remediation and Restoration of Contaminated Nuclear and Norm Sites* 5–276 (Elsevier Science, 2015).

[11] Hannan M, Wahid K, Nguyen N (2015). Assessment of natural and artificial radionuclides in Mission (Texas) surface soils. J. Radioanal. Nucl. Chem..

[12] Szerbin P, Koblinger-Bokori E, Koblinger L, Végvári I, Ugron A (1999). Caesium-137 migration in Hungarian soils. Sci. Total Environ..

[13] Pumpanen J (2016). 137Cs distributions in soil and trees in forest ecosystems after the radioactive fallout – Comparison study between southern Finland and Fukushima, Japan. J. Environ. Radioact..

[14] Sandeep S, Manjaiah KM, Sachdev P, Sachdev MS (2009). Effect of nitrogen, potassium and humic acid on 134Cs transfer factors to wheat from tropical soils in Neubauer growth units. Environ. Monit. Assess..

[15] Mesrar H (2017). Vertical and lateral distribution of fallout 137Cs and soil properties along representative toposequences of central Rif, Morocco. J. Environ. Radioact..

[16] Al-Sulaiti H (2016). Determination of 137Cs activity in soil from Qatar using high-resolution gamma-ray spectrometry. Radiat. Phys. Chem..

[17] Shomar B, Amr M, Al-Saad K, Mohieldeen Y (2013). Natural and depleted uranium in the topsoil of Qatar: Is it something to worry about?. Appl. Geochem..

[18] Navas A, Gaspar L, López-Vicente M, Machín J (2011). Spatial distribution of natural and artificial radionuclides at the catchment scale (South Central Pyrenees). Radiat. Meas..

[19] Wang Q (2016). Environmental evolution records reflected by radionuclides in the sediment of coastal wetlands: A case study in the Yellow River Estuary wetland. J. Environ. Radioact..

[20] Chandrasekaran A (2015). Assessment of natural radioactivity and function of minerals in soils of Yelagiri hills, Tamilnadu, India by Gamma Ray spectroscopic and Fourier Transform Infrared (FTIR) techniques with statistical approach. Spectrochim. Acta A.

[21] Tsai T, Liu C, Chuang C, Wei H, Men L (2011). The effects of physico-chemical properties on natural radioactivity levels, associated dose rate and evaluation of radiation hazard in the soil of Taiwan using statistical analysis. Budapest, Hungary. J. Radioanal. Nucl. Chem..

[22] Walther, C. & Gupta, D. K. *Radionuclides in the Environment: Influence of chemical speciation and plant uptake on radionuclide migration* (Springer, 2015).

[23] Al-Sulaiti H (2010). A preliminary report on the determination of natural radioactivity levels of the State of Qatar using high-resolution gamma-ray spectrometry. Nuclear Instruments and Methods in Physics Research Section A: Accelerators, Spectrometers, Detectors and Associated Equipment.

[24] Sabljic, A. *Environmental and ecological chemistry*. (Oxford, U.K., Eolss, 2009).

[25] Poinssot, C. & Geckeis, H. Overview of radionuclide behaviour in the natural environment. *Radionuclide Behaviour in the Natural Environment*. 1–10 (2012).

[26] Kumar A (2011). Distribution, enrichment and principal component analysis for possible sources of naturally occurring and anthropogenic radionuclides in the agricultural soil of Punjab state, India. Radiat. Prot. Dosim..

[27] UNSCEAR. U (2000). Nations Scientific Committee on the Effects of Atomic Radiation. Sources and Effects of Ionizing Radiation. Report to the General Assembly, Annex A & B.

[28] Bajoga A, Alazemi N, Shams H, Regan P, Bradley D (2016). Evaluation of naturally occurring radioactivity across the State of Kuwait using high-resolution gamma-ray spectrometry. Radiat. Phys. Chem..

[29] Forkapic S (2017). Correlations between soil characteristics and radioactivity content of Vojvodina soil. J. Environ. Radioact..

[30] Application of the Concepts of Exclusion, Exemption and Clearance In Safety Standards Series No. RS-G-1.7. International Atomic Energy Agency (IAEA) (2004).

[31] Karataşlı M, Turhan Ş, Varinlioğlu A, Yeğingil Z (2016). Natural and fallout radioactivity levels and radiation hazard evaluation in soil samples. Environ. Earth Sci..

[32] Dragović S, Janković-Mandić L, Dragović R, Đorđević M, Đokić M (2012). Spatial distribution of the 226Ra activity concentrations in well and spring waters in Serbia and their relation to geological formations. J. Geochem. Explor..

[33] Al-Sulaiti H, Nasir T, Regan PH, Bradley D (2014). Effect of the grain size of the soil on the measured activity and variation in activity in surface and subsurface soil samples. Pak. J. Sci. Ind. Res. Ser. A: Phys. Sci..

[34] Skytte-Jensen, B. The geochemistry of radionuclides with long half-lives: their expected migration behavior 5-52 (Springfield: National Technical Information Service, 1980).

[35] Karadeniz Ö, Çakır R, Karakurt H (2015). Estimation of vertical migration velocity of 137Cs in the Mount IDA/Kazdagi, Turkey. J. Environ. Radioact..

[36] Akkaya G, Kaynak G, Kahraman A, Gurler O (2012). The investigation of radionuclide distributions in soil samples collected from Bursa, Turkey. Radiat. Prot. Dosim..

[37] Al-Sulaiti H (2012). Determination of the natural radioactivity levels in north west of Dukhan, Qatar using high-resolution gamma-ray spectrometry. Appl. Radiat. Isot..

[38] Al-Azemi N, Bajoga A, Bradley D, Regan P, Shams H (2016). Soil radioactivity levels, radiological maps, and risk assessment for the state of Kuwait. Chemosphere..

[39] Alshahri F (2017). Radioactivity of 226 Ra, 232 Th, 40 K and 137 Cs in beach sand and sediment near to desalination plant in eastern Saudi Arabia: Assessment of radiological impacts. J. King Saud Univ. Sci..

[40] Bajoga. AD, Alazemi N, Regan PH, Bradley DA (2015). Radioactive investigation of NORM samples from Southern Kuwait soil using high-resolution gamma-ray spectroscopy. Radiat. Phys. Chem..

[41] Al-Kinani AT, Amr MA, Al-Saad KA, Helal AI, Al-Dosari MM (2012). Radioactivity measurements and risk assessments in soil samples at south and middle of Qatar. Arab J. Nucl. Sci. Appl..

[42] Al-Hamarneh I, Awadallah M (2009). Soil radioactivity levels and radiation hazard assessment in the highlands of northern Jordan. Radiat. Meas..

[43] Abojassim A, Oleiwi M, Hassan M (2016). Natural radioactivity and radiological effects in soil samples of the main electrical stations at Babylon governorate. University of Babylon, College of Education for Pure Science, Department of Physics. Iraq.

[44] ICRP. Recommendations of the International Commission on Radiological Protection **212**(1–3) (1990). 2053748

[45] UNSCEAR. Sources and effects of ionizing radiation. United Nations Scientific Committee on the effects of Atomic Radiation. Report to the general Assembly, United Nations, New York. Annex A, 18–33 (1993).

[46] Altıkulaç, A., Turhan, Ş. & Gümüş, H. Activity concentration of terrestrial and anthropogenic radionuclides (226Ra, 222Rn, 232Th, 40K, and 137Cs) in soil samples. *Environ*. *Earth Sci*. **75****(****1****)** (2015).

[47] Ibikunle SB, Ajayi OS, Arogunjo AM (2013). Effect of Geology on Soil Radioactivity and Risks to Humans Based on Data from Several Towns in Nigeria. Environ. Forensics..

[48] El-Mageed AA (2011). Assessment of natural and anthropogenic radioactivity levels in rocks and soils in the environments of Juban town in Yemen. Radiat. Phys. Chem..

[49] L’Annunziata, M. F. *Handbook of radioactivity analysis* (San Diego Academic Press, 2003).

[50] Al-Qaradawi I (2015). Radioactivity Levels in The Marine Environment Along the Exclusive Economic Zone (EEZ) Of Qatar. Mar. Pollut. Bull..

[51] Choppin, G., Liljenzin, J., Jan, R. & Christian, E. *Radiochemistry and Nuclear Chemistry Fourth-Edition* (Elsevier, 2013).

[52] Lin W (2015). Radioactivity impacts of the Fukushima Nuclear Accident on the atmosphere. Atmos. Environ..

[53] Jwanbot, D. *Radio Levels in Soil and Food Samples in Mining Areas on the Jos-Plateau* (Lap Lambert Academic Publishing, 2012).

